# Errata

**Published:** 2015-07-03

## Vol. 64, No. 19

In the report, “Fatal and Nonfatal Drowning Outcomes Related to Dangerous Underwater Breath-Holding Behaviors — New York State, 1988–2011,” errors occurred. The author list and author affiliations should read as follows:

Christopher Boyd^1^; Amanda Levy, MSPH^1^; Trevor McProud, MS^1^; **Li** Huang, PE^1^; Eli Raneses, MPH^1^; Carolyn Olson, MPH^1^; **Eric Wiegert, MPH^2^** (Author affiliations at end of text)

^1^Division of Environmental Health, New York City Department of Health and Mental Hygiene**;** **^2^Bureau of Community Environmental Health and Food Protection, New York State Department of Health**.

In addition, on page 520, in the second paragraph, the fourth and fifth sentences should read:

“Fifteen of the 16 incidents in this case study occurred at **New York state** bathing facilities that require an operating permit from **their local health department, or under the oversight of the New York State Office of Parks, Recreation and Historic Preservation. All incidents had witnesses who reported predrowning behaviors.** However, research suggests that more than half of drowning incidents are not witnessed (9,10).”

Finally, the following acknowledgments should be included:

“**Douglas Sackett, Timothy Shay, Amanda Tarrier, Bureau of Community Environmental Health and Food Protection, New York State Department of Health. Regional office and local health department staff members throughout New York state.**”

## Vol. 64, No. 23

In the report, “Opioid Overdose Prevention Programs Providing Naloxone to Laypersons — United States, 2014,” an error occurred. On page 633, in the second full paragraph, the fifth sentence should read: “A total of 111,60**7** vials (79.7%) of injectable naloxone (21.4% 10 mL and 58.1% 1 mL) and 28,446 (20.3%) vials of intranasal naloxone were provided to laypersons.”

